# Electromyographic Assessment of the Extrinsic Laryngeal Muscles: Pilot and Descriptive Study of a Vocal Function Assessment Protocol

**DOI:** 10.3390/s25206430

**Published:** 2025-10-17

**Authors:** Jéssica Ribeiro, André Araújo, Andreia S. P. Sousa, Filipa Pereira

**Affiliations:** 1 Escola Superior de Saúde, Instituto Politécnico do Porto, Rua Dr. António Bernardino de Almeida, 4200-072 Porto, Portugal; jessicabarbosa.tf@gmail.com (J.R.); 10180670@ess.ipp.pt (F.P.); 2 Centro de Investigação em Reabilitação (CIR), Escola Superior de Saúde, Instituto Politécnico do Porto, Rua Dr. António Bernardino de Almeida, 4200-072 Porto, Portugal; andrearaujo@ess.ipp.pt

**Keywords:** surface electromyography, extrinsic laryngeal muscles, voice, voice assessment, speech therapy

## Abstract

Aim: The aim of this study was to develop and test a surface electromyography (sEMG) assessment protocol to characterise the activity of the extrinsic laryngeal muscles (suprahyoid and infrahyoid) during phonatory tasks and vocal techniques. Methodology: The protocol of assessment was based on electromyographic assessment guidelines and on clinical voice evaluation needs and was tested in six healthy adults with no vocal disorders. Surface electromyographic activity of suprahyoid and infrahyoid muscles was acquired during different reference tasks (rest, reading, maximum contractions) and six vocal tasks, including nasal sounds, fricatives, and semi-occluded vocal tract exercises. A laryngeal accelerometer was used for detecting the beginning and end of each exercise. The average activity during each task was normalised by the signal obtained in the incomplete swallowing task for the SHM and by the sniff technique for the IHM. Results: The range of activation values varied across tasks, with higher percentages observed in plosive production and in the “spaghetti” technique, while nasal and fricative sounds tended to show lower activation values within the group. A consistent pattern of simultaneous activation of suprahyoid and infrahyoid muscles was observed during phonation. Conclusions: The protocol proved potential for clinical application in speech–language pathology as it enabled the characterisation of muscle activity in determinant muscles for vocal function. Larger samples and further validation of the time-marking system are needed. This study provides a foundation for integrating sEMG measures into functional voice assessment.

## 1. Introduction

Surface electromyography (sEMG) has proven to be an increasingly relevant tool for assessing the muscles involved in orofacial functions and is particularly integrated into the study of the stomatognathic system (SS) [1]. This system comprises a set of fundamental anatomical structures (such as the facial muscles, tongue, mandible, pharynx, and larynx) for vital functions such as chewing, swallowing, breathing, voice and speech [2].

Clinical assessment of the structures and functions of the SS is carried out by various professionals, including speech therapists. At this level, clinical assessment is still heavily based on perceptual methods [3], increasing its subjectivity and consequently limiting the reliability and accuracy of the diagnosis. Therefore, the need to incorporate objective and instrumental methods becomes clear, promoting evidence-based practice and improving the quality of therapeutic intervention. sEMG stands out as a non-invasive method to measure muscle electrical activity, making it a valuable technique for functional analysing the muscles involved in the functions of the SS [4,5].

Despite the potential of sEMG, there is a significant gap in the standardisation of specific protocols for its application in the field of speech therapy [6]. However, other areas of the SS remain underexplored, requiring systematised guidelines for their use. This limitation jeopardises the scope and clinical usefulness of the technique, especially in complex areas such as voice. sEMG has already been explored in the voice field, although mainly applied to different muscles. Several studies have used sEMG to investigate intrinsic laryngeal muscle activity, particularly in pathological contexts such as vocal fold paralysis, where needle sEMG has been the method of choice [7]. Research has also extended to orofacial and cervical musculature, such as the orbicularis oris and lip-closing muscles, demonstrating the potential of sEMG both for assessment and as a guide in functional training exercises [1,8]. These findings support the view that sEMG, although still not standardised for vocal assessment, can provide objective and clinically relevant data to complement traditional perceptual approaches.

There are already a few objective methods available in this area (such as electroglottography, videostroboscopy and acoustic analysis), and, as said, sEMG is used mainly to study the activity of the intrinsic muscles of the larynx, in cases where neurological activity is compromised, as in laryngeal paralysis [6]. However, it requires depth electromyography, an invasive method. Although this technique provides relevant information on vocal production, its limitation lies in the fact that vocal function is not solely dependent on the intrinsic activity of the larynx, but results from the coordinated interaction of various structures, including the suprahyoid (SHM) and infrahyoid (IHM) muscles, which make up the extrinsic musculature of the larynx [9,10]. These muscles play a crucial role in stabilising and mobilising the larynx during phonation, directly influencing vocal quality [9].

In 2024, a study by Hong et al. stated that 11.71 per cent of adults suffer from dysphonia [11]. According to Marchese et al. (2022), dysphonia is more common in females. In addition, both organic dysphonia and functional dysphonia show prevalence rates that are not statistically significant, making it necessary to create objective assessment methods for each type [12]. sEMG has been used, not only with assessment purposes, but also as a therapy resource in other areas of the SS. It has been concluded that it can be used in breathing intervention as a guide for functional muscle training [8]. It was found to be a relevant tool for monitoring exercises, in order to understand if muscle recruitment in isotonic activities was the most appropriate for the intended exercise [13].

In this context, there is a clear need to further study the extrinsic muscles of the larynx, not only as a support for vocal production, but also as an active element and modulator of vocal function. Some studies suggest that the use of sEMG applied to these muscles represent a promising way to improve voice assessment, adding more objective, systematic, and well-founded data [14,15]. As shown in breathing, it could also be adapted to monitor muscle activity during therapy exercises. However, the scarcity of specific protocols for this application limits its dissemination, effectiveness, and clinical applicability, highlighting the urgent need to develop standardised and tested methodologies [14].

The aims of this study are therefore (a) to develop a procedural protocol for the kinematic assessment of extrinsic laryngeal muscles to characterise vocal function; (b) to test the procedures for applying this protocol in an experimental context; and (c) to describe the electromyographic activity of the supra- and infrahyoid muscles in different phonatory tasks and vocal techniques.

## 2. Materials and Methods

### 2.1. Subjects

In this exploratory study, six volunteers representative of the adult population without vocal pathology took part, recruited through a convenience sampling process. Participants aged between 19 and 23 were included. Exclusion criteria were the existence of a previous or current diagnosis of vocal pathology, signs or symptoms of skin problems that could interfere with data collection through sEMG and being enrolled in a speech therapy course. The inclusion and exclusion criteria were selected with the aim of selecting a sample that was closer to the reality of the population and with a mastery of voice with little differentiation in technique.

The study was approved by the Ethics Committee of the Superior School of Health from Porto Polytechnic Institute. All the participants were able to freely decide whether to take part in the study by signing an informed consent form.

### 2.2. Experimental Procedures

The room where the data collection took place was selected considering the absence of noise and visual disturbances. Each participant was prepared for the data collection phase in a maximum of 10 min. Each participant remained seated on a chair, with their torso upright, their feet flat on the floor with their legs at 90°, their arms resting on their legs and their eyes open, looking straight ahead. Preparation for electrode placement included first tracing the application sites and then exfoliating the skin with a scrub and alcohol to reduce impedance. In men, the area had to be shaved to improve contact, using disposable material. After data collection, the skin was moisturised, and the participants were given aftercare instructions. The impedance measurement ensured that the abrasion was adequate. The exact location of the electrodes was marked with a make-up pencil and based on specific anatomical references (through observation and palpation) to capture signals from the SHM and IHM, with a ‘ground’ electrode placed on the right clavicle. The order of placement followed the sequence SHM, IHM and ground, respecting SENIAM recommendations to minimise interference. The accelerometer (Plux wireless biosignals S.A, Lisboa, Portugal) was then attached to the superficial area of the larynx. The accelerometer was used as an essential auxiliary tool for validating and analysing the data collected using sEMG. This device was placed at the level of the larynx to detect vertical movements during phonation tasks. The main function of the accelerometer was to accurately identify the start and end time of each task by analysing the trace of its signal on the *Z* axis (vertical movement). The start and end of each vocal task were determined manually by visually inspecting the signal trace on the *Z* axis of the accelerometer, which allowed for precise delimitation of the time period for analysis of the electromyographic signal.

The accelerometer thus made a decisive contribution to the normalisation and interpretation of electromyographic data, ensuring a more rigorous and objective analysis of muscle dynamics during vocal production.

Each participant adopted a habitual posture with minimal muscle activity for 10 s to record baseline activity. Three more reference behaviours were also requested: maximum muscle contraction of the IHM (using the sniff technique), maximum muscle contraction of the SHM (incomplete swallowing task) and reading text. Electromyographic signal normalisation was performed differently for the two muscle groups. For the SHM, the incomplete swallowing task was used as a reference for maximum contraction, as suggested in the literature [16,17]. In the case of the IHM, there is no consensus on the most appropriate task to induce maximum contraction. Thus, this pilot study tested two alternatives described in previous studies and in clinical practice: the sniff technique and the spaghetti technique. The comparison of the results obtained with both techniques allowed us to evaluate their feasibility as reference tasks for normalising the IHM signal, although further studies with larger samples are needed to confirm their validity.

Next, six activities were conducted: (i) spaghetti technique; (ii) upward glissando; (iii) prolonged /b/ technique; (iv) nasal sound technique; (v) fricative sound production technique; (vi) plosive sound technique. The six vocal tasks were selected because they reflect common techniques in voice therapy, covering sounds with low intraoral pressure (nasals, fricatives) to tasks with greater muscular demand (plosives, prolonged/b/, ascending glissando, and the “spaghetti” technique), allowing for the assessment of different functional patterns relevant in a clinical context.

### 2.3. sEMG

The activity of the larynx muscles was assessed by surface electromyography. The electromyographic signal of these muscles was monitored using a bioPLUX research wireless signal acquisition system (Plux wireless biosignals S.A., Lisboa, Portugal). The signals were collected at a sampling frequency of 1000 Hz and were preamplified in each electrode and then fed into a differential amplifier with an adjustable gain setting (25–500 Hz; common-mode rejection ratio: 110 dB at 50 Hz, input impedance of 100 MΩ and gain of 1000). Self-adhesive silver chloride electromyographic electrodes were used in a bipolar configuration with a distance of 20 mm between detection surface centres. The skin impedance was measured with an electrode impedance checker. The electromyography signals were analysed with the Acqknowledge software (version 3.9; Biopac Systems, Inc., Goleta, CA, USA).

A specific protocol was created to collect electromyographic data to assess the kinematics of the extrinsic laryngeal muscles during phonatory tasks and vocal techniques. This protocol was adapted from previous models, validated by a panel of experts (content validity), and tested with an accelerometer (criterion validity). Pre-tests with three volunteers allowed for adjustments to the procedures and tasks. Considering the size and positioning of the extrinsic muscles of the larynx, and based on the tests conducted, it was decided to monitor muscle groups rather than isolated muscles. The protocol was structured in such a way as to collect two signals: one for the SHM group, namely the digastric and mylohyoid muscles, and another for the IHM group, made up of the sternohyoid, thyrohyoid and omohyoid muscles. The bipolar electrodes were 2 cm apart and placed as shown in the following image.

**Figure 1:**
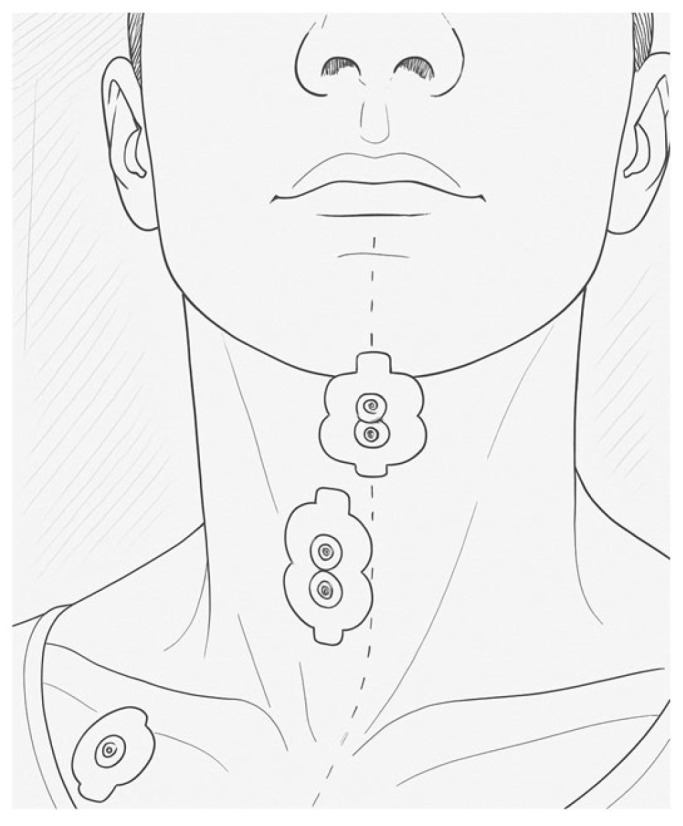
Representation of the electrode position on SHM and IHM. The dot line represents the midline.

The electrodes were placed following specific anatomical landmarks for each muscle group, as shown in Figure 1. For the SHM, an electrode was placed longitudinally in the submandibular region to capture the electromyographic signal from this muscle group. The exact location was selected by bone palpation.

For the IHM, the electrode was placed 1 centimetre lateral to the midline of the neck, traced from the upper margin of the hyoid bone to the jugular notch, to capture the activity of the IH muscle group. The reference (“ground”) electrode was placed on the right clavicle, the side chosen to minimise heartbeat capture. The recommended order for placing the electrodes is as follows: SH, IH, and “ground” electrode.

### 2.4. Data Analysis and Statistical Analysis

After analysing the quality of the signal, the data was processed. The signal was filtered withan IIR-Bandpass digital filter with a frequency between 20 and 500 Hz and the Root Mean Square was calculated in a sliding window of 100 ms. The start and end of each task was determined based on the accelerometer’s trace on the vertical (*z*) axis, to identify the periods of activity of the SHM and IHM muscle groups. From these intervals, the average percentage of the electrical activity of each group in relation to its maximum activity was calculated. Analysis of the electromyographic signal is based on data normalisation, which can refer to baseline activity or maximum contraction. In this exploratory study, the average was used, with speech as the reference task because it is functionally representative and comparable between muscle groups. Data processing and descriptive analysis (calculation of averages and percentages) were performed using Microsoft Office Excel 2007 (Microsoft Corporation, Redmond, WA, EUA).

## 3. Results

The data presented is expressed in percentage values, based on 100% of the maximum muscle activity (MMA) of each muscle group, baseline activity (BA) and the speech/reading task (mean value), respectively, and standard deviations are indicated in the graphs as error bars.

**Figure 2:**
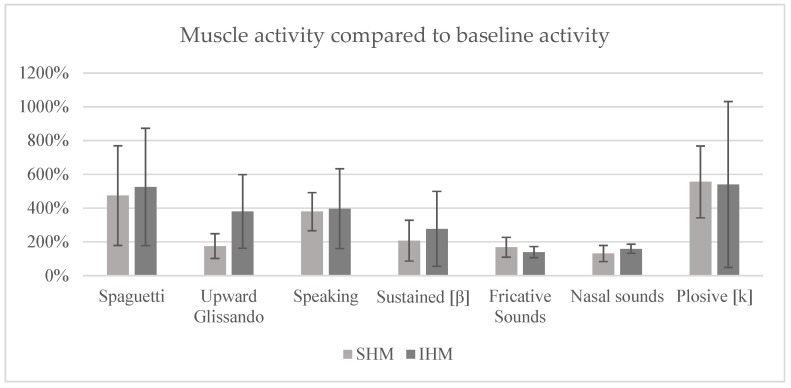
Mean percentage of muscle activity compared to baseline activity (error bars = standard deviation).

Based on the data observed, it can be seen that, when reading the signal in relation to AB (Figure 2), the technique that promoted the greatest recruitment of muscle activity in both muscle groups was the production of plosives, with values of 555% for SHM and 540% for IHM. The “spaghetti” technique had a recruitment rate of 474% for the SHM and 525% for the IHM, while the prolonged/b/technique showed 469% in the SHM and 359% in the IHM. The speech task resulted in 379% activation in the SHM and 396% in the IHM. The upward glissando technique registered 175% for SHM and 380% for IHM. Finally, the techniques for producing fricative and nasal sounds were the ones that elicited the least muscle activation, with values of 168% and 139% for SHM and IHM, respectively, in the fricative technique, and 131% and 159% for SHM and IHM, respectively, in the nasal technique.

**Figure 3:**
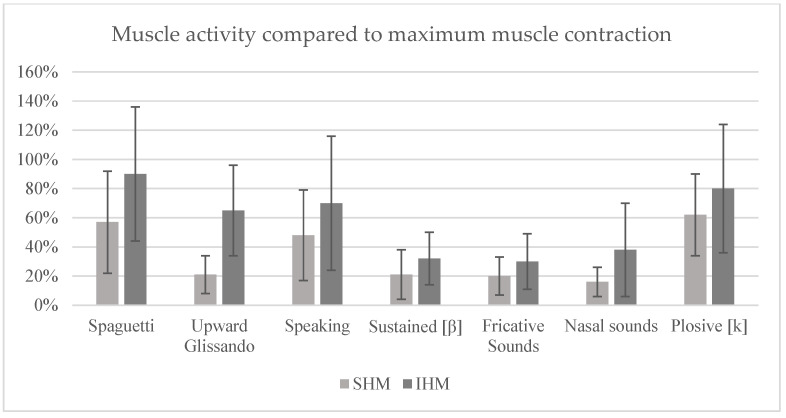
Mean percentage of muscle activity compared to maximum muscle contraction (error bars = standard deviation).

Analysing the signal in relation to the MMA (Figure 3) reveals that the technique that promoted the greatest activation of the SHM was the prolonged/b/, with 86%, while the ‘Spaghetti’ technique showed the greatest recruitment of the IHM, with 90%. The same techniques also showed 80% activation in the IHM and 57% in the SHM, respectively. The plosive production technique resulted in 62 per cent recruitment in the SHM and 81 per cent in the IHM. In the case of the speech task, 48 per cent muscle activation was recorded in the SHM and 71 per cent in the IHM. The upward glissando technique showed 21 per cent recruitment in the SHM and 65 per cent in the IHM. Finally, the techniques for producing fricative and nasal sounds were again the ones that induced the least muscle activity, with 21% and 16% in SHM and 31% and 38% in IHM, respectively.

**Figure 4:**
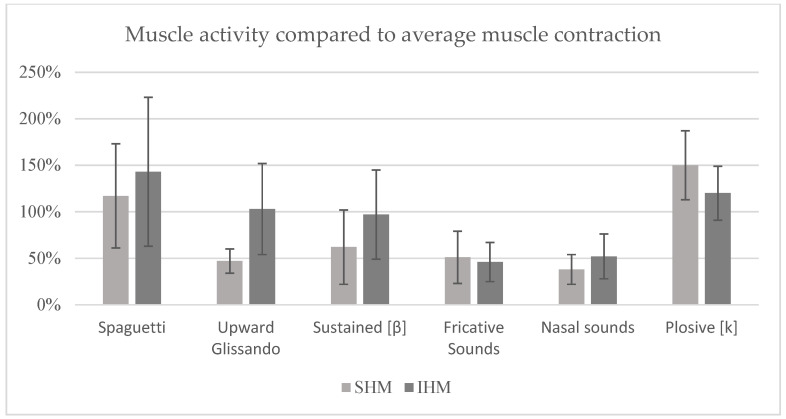
Mean percentage of muscle activity compared to average muscle contraction (reading activity) (error bars = standard deviation).

Analysing the signal in relation to the speech task (mean value) (Figure 4), it was found that the technique with the highest recruitment of the SHM was the production of plosive sounds, with 150%, while the ‘Spaghetti’ technique showed the highest activation of the IHM, with 143%. These same techniques also showed 120% recruitment in the IHM and 118% in the SHM, respectively. The prolonged/b/technique resulted in 127% activation in the SHM and 97% in the IHM. The ascending glissando technique showed 47% activation in the SHM and 103% in the IHM. As in the previous analyses, the techniques for producing fricative and nasal sounds were the ones that elicited the least muscle recruitment, with values of 51% and 38% in SHM and 46% and 52% in IHM, respectively.

## 4. Discussion

The literature describes the SHM as mainly responsible for laryngeal and hyoid bone elevation, and they tend to be more active in tasks that involve pitch modulation. The IHM, on the other hand, is commonly associated with stabilising and lowering the larynx, acting continuously to support laryngeal positioning during activities such as speaking and singing. Within this framework, the results obtained in this pilot study reveal trends that warrant reflection, particularly considering the exploratory nature of the protocol.

The testing phase of the procedures was crucial for protocol development, especially in defining reference tasks for signal normalisation. For the SHM, the incomplete swallowing task remains the most consolidated approach in the literature [14,15]. For the IHM, however, there is no consensus, which justifies the decision to compare the sniff and spaghetti techniques. In this study, there was a tendency for the sniff technique to elicit higher IHM activation, although doubts remain as to whether it effectively represents a true maximum contraction. This issue highlights the importance of carefully selecting reference tasks, since the choice of normalisation method can directly affect data interpretation.

When considering the speech task, the results indicated a relatively balanced tendency of activation between SHM and IHM, reinforcing the clinical relevance of using this task as a functional reference condition. The observation that both groups contributed to the stabilisation of the larynx and hyoid bone during speech underscores the importance of analysing these muscles within contexts that reflect everyday vocal behaviour.

The analysis of the ascending glissando task showed a trend towards higher IHM activation, contrary to what would be expected for SHM given their role in pitch modulation. This apparent discrepancy may reflect the contribution of factors beyond direct muscular action, such as the effect of subglottic pressure during high-pitched phonation. Rather than contradicting previous studies, this finding reinforces the need to contextualise results within the methodological conditions of this pilot protocol and highlights the complexity of extrinsic muscle involvement in vocal function.

Semi-occluded vocal tract tasks elicited distinct trends depending on the articulatory configuration. Plosives and the prolonged [b] tended to recruit both muscle groups more strongly, a pattern that may be linked to the increased intraoral pressure requiring laryngeal stabilisation. From a clinical perspective, these tasks may be relevant when the goal is to promote greater muscle engagement, such as in hypokinetic dysphonia. Conversely, tasks involving fricatives and nasals showed lower recruitment tendencies, consistent with their lower airflow resistance. These may therefore be more appropriate in early stages of rehabilitation or in conditions associated with excessive muscle activity. It should be stressed that these are descriptive tendencies, not statistical differences.

The spaghetti technique showed a trend towards marked IHM activation, accompanied by SHM recruitment, which may relate to the combined action of lowering and stabilising forces on the larynx. This observation illustrates the complexity of muscular synergies during tasks that induce anatomical and physiological changes, and reinforces the relevance of including different types of tasks when testing clinical applicability.

The comparison of reference conditions (baseline, MMA, and speech task) highlighted the importance of the chosen normalisation method. Baseline produced higher variability, limiting comparability between participants. The MMA reference provided more moderate percentages, but methodological limitations in eliciting true maximum contraction of the IHM may have led to an overestimation of relative activity. By contrast, normalisation to the speech task tended to yield more balanced results, reflecting a functional condition that may be more clinically meaningful. This finding supports the use of speech as a reference for capturing physiologically relevant patterns in extrinsic muscle activity.

Overall, the results should be understood as preliminary tendencies rather than definitive conclusions. Their value lies mainly in demonstrating the feasibility of the protocol and in highlighting methodological aspects that require further investigation, particularly the standardisation of normalisation strategies. From a clinical perspective, the inclusion of different types of tasks and reference conditions appears to be a promising approach to better capture the functional role of extrinsic laryngeal muscles in voice production. Future studies with larger samples and inferential statistical analysis will be essential to validate these trends and to confirm their potential clinical applications.

## 5. Conclusions

This pilot study developed and applied a functional protocol for the electromyographic assessment of extrinsic laryngeal muscles in vocal tasks, indicating its potential relevance to the clinical practice of speech therapists. The use of sEMG proved to be a viable tool for collecting objective data on the activation of the supra- and infrahyoid muscles, with the potential to complement traditional subjective methods and support more informed therapeutic decisions.

The results show that the protocol is applicable in a real-world context and makes it possible to characterise muscle activation patterns in different vocal techniques. There was a tendency for tasks such as plosive sounds and the sound [b] to elicit higher activation values, which may be clinically relevant in the context of hypokinetic dysphonia. Conversely, fricatives and nasals tended to show lower values, which may be more adequate in preliminary stages of rehabilitation or in hyperfunctional dysphonia. These are descriptive tendencies that require confirmation in future studies.

However, the study has limitations, namely the need to further validate the use of the accelerometer as a temporal reference. Although it was useful in delimiting the tasks and did not interfere with the mobility of the structures, its use could be further developed in future studies, ideally in conjunction with acoustic signal analysis. Limitations related to the small number of participants and the age range restricted to young adults (19–23 years) reinforce the need for future investigations with larger and more diverse samples to increase the validity and generalisability of the results.

Although the study demonstrated the feasibility and clinical potential of the protocol, we recognise that the uncertainty in the literature regarding the ideal task for MMA of the IHM represents an important limitation. This lack of methodological consensus may directly impact the interpretation of results, especially those presented as a percentage of Maximum Muscle Activity and reinforces the need for future research to further investigated usage and validate this aspect. Our exploratory approach, by testing different standardisation methodologies, aims precisely to contribute to the discussion and guide the choice of a more robust and standardised method in subsequent studies.

Future studies should include a larger and more diverse sample, integrate other muscles of the stomatognathic system and explore in more detail exercises that deviated from what was expected, such as ascending glissando. Combining sEMG with other objective tools could strengthen the clinical validity of the protocol, contributing to a more precise, individualised and evidence-based vocal assessment. Additionally, we emphasise that future investigations with larger samples should incorporate inferential statistical analyses (such as ANOVA or non-parametric equivalents) to strengthen the interpretation of results and allow for generalisations.

## Data Availability

The data presented in this study are available from the corresponding author upon reasonable request due to ethical restrictions related to participant confidentiality.

## Abbreviations

The following abbreviations are used in this manuscript:

sEMG	Surface Electromyography
SS	Stomatognathic System
IHM	Infrahyoid Muscles
SHM	Suprahyoid Muscles
